# The spliceosome as target for anticancer treatment

**DOI:** 10.1038/sj.bjc.6604801

**Published:** 2008-11-25

**Authors:** R J van Alphen, E A C Wiemer, H Burger, F A L M Eskens

**Affiliations:** 1 Department of Medical Oncology, Erasmus University Medical Centre, PO Box 2040, Rotterdam 3000 CA, the Netherlands

**Keywords:** spliceosome, alternative splicing, planiedolide, E-7107, spliceostatin A

## Abstract

The spliceosome is a ribonucleoprotein complex involved in RNA splicing, that is, the removal of non-coding introns from precursor messenger RNA. (Alternative) Splicing events may play an essential role in tumourigenesis. The recent discovery that the spliceosome is a target for novel compounds with anticancer activity opens up new therapeutic avenues.

Most protein-coding genes in our genome are composed of multiple exons interrupted by introns. The exons are usually relatively short about 50–250 base pairs, whereas introns are much larger and can be up to several thousands of base pairs. Together the exon sequences encode the protein, whereas the introns are generally non-coding. When a gene is transcribed, exons and intron sequences are converted into a single precursor messenger RNA (pre-mRNA). In a process called splicing, the intron sequences are removed from the pre-mRNA and the exons fused together resulting in the formation of the mature messenger RNA (mRNA), which is subsequently capped at its 5′ end and polyadenylated at its 3′ end, and transported out of the nucleus to be translated into protein in the cytoplasm. In general, most genes give rise to multiple spliced transcripts by alternative splicing. These transcripts contain different combinations of exons, and sometimes introns or part thereof, leading to different mRNA variants and the synthesis of alternative proteins (Figure 1). The mere existence of alternatively spliced products greatly increases cellular and organismal complexity and may have allowed for evolution by producing additional regulation and diversification of gene function. It is obvious that splicing and alternative splicing must be tightly regulated and executed in time and space not to interfere with the normal cellular and organismal physiology. The spliceosome, an intracellular complex of multiple proteins and ribonucleoproteins, is the main cellular machinery guiding splicing. Recently, two natural compounds interfering with the spliceosome were found to display antitumour activity *in vitro* and *in vivo*. Therefore, it is conceivable that inhibiting the spliceosome could serve as a novel target for anticancer drug development (Kaida *et al*, 2007; Kotake *et al*, 2007).

## Splicing and alternative splicing: the role of the spliceosome

**Figure 2:**
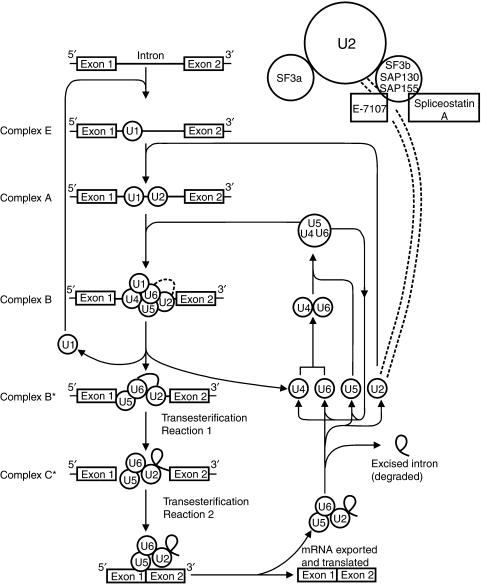
The spliceosome assembly cycle. Through interactions with various proteins that recognise specific splice site features, the spliceosome components, that is, small nuclear ribonucleoproteins (snRNPs) designated with U1, U2, U4, U5 and U6 are sequentially recruited to the splice site and assembled into the spliceosome. Once completed, splicing is catalysed in two consecutive transesterification reactions. In the initial step, the 2′ OH group of the branch point adenosine upstream of the 3′ end of the intron reacts with the 5′ splice junction, forming a novel 2′, 5′ phosphodiester bond between the branch point and the 5′ terminal nucleotide of the intron, giving rise to a lariat structure. In the second reaction, the 3′ OH of the 5′ exon attacks the 3′ splice junction producing linked 5′ and 3′ exons and liberating the intron. Subsequently, the snRNPs involved are released and recycled in the splicing process. In the top right-hand corner, a detailed view of U2 snRNP with subcomplexes, SF3a and SF3b, is shown. The spliceosome-associated proteins (SAP) 130 and/or 155 are targeted by pladienolide derivatives (a.o. E-7107) and spliceostatin A.

To direct correct processing of pre-mRNA, the intron sequences contain a number of core splicing signals, notably the conserved splice-site sequences at their extreme 5′ and 3′ ends and a conserved branch point region. The branch point sequence and the branch point itself, usually an adenosine, are located about 20–40 nucleotides upstream of the 3′ splice site. The critical process of recognising splice sites and the removal of introns and/or exons is the task of the spliceosome (Staley and Guthrie, 1998; Will and Lührmann, 2001). The spliceosome consists of five non-coding uridine-rich small nuclear ribonucleoproteins (U snRNPs) and a multitude of associated proteins, creating a network of RNA–RNA, RNA–protein and protein–protein interactions (Zhou *et al*, 2002; Jurica and Moore, 2003) (Figure 2). In fact, well over 200 different splicing factors interacting with the spliceosome have been identified, and it is clear that much more research is needed before we fully understand all the intricacies of RNA splicing (Jurica and Moore, 2003; Wang and Burge, 2008). The various spliceosome components, in particular, the small nuclear RNPs, are sequentially recruited to the splice sites. Next, they are assembled into the spliceosome after which splicing is initiated by a series of two transesterification reactions producing two ligated exons and a liberated intron (Staley and Guthrie, 1998; Wang and Burge, 2008) (Figure 2). Splicing at different locations in the same pre-mRNA transcript, alternative splicing, enables a gene to produce variant mature mRNAs, and consequently functionally different proteins (House and Lynch, 2008). The precise mechanisms of and/or triggers for alternative splicing are yet unknown. In higher eukaryotes, like humans, the accuracy of splicing is not solely dictated by base-pairing interactions of the U snRNPs with the pre-mRNA. Owing to degeneracy and poor definition of small splice sites at the end of long introns, splice-site recognition is also influenced by (i) the coupling of splicing with other processes such as transcription, (ii) the velocity of the splicing reaction, (iii) external stimuli, like the presence of growth factors or oxidative stress and (iv) the three-dimensional structure of pre-mRNA itself (Tazi *et al*, 2005b; Disher and Skandalis, 2007; House and Kristen, 2008). In the latter case, the three-dimensional folding of pre-mRNA determines whether a (in pre-mRNA embedded) splice regulator will be located in the vicinity of a splice site.

Four regulatory sequence elements have been identified; two stimulative ones, splicing enhancers located in introns (ISE) or exons (ESE), and two suppressive ones, splicing silencers located in introns (ISS) or exons (ESS), respectively (Tazi *et al*, 2005b; House and Kristen, 2008 and references therein). The enhancers generally recruit spliceosome components or splicing factors to a splice site on pre-mRNA. On the other hand, the splicing silencers block specific splicing sites; hence interfere with the binding of components of the spliceosome with the blocked splice site on pre-mRNA. These specific exonic and intronic pre-mRNA sequences are modulated by interactions with non-spliceosomal nuclear RNA-binding proteins. Both repressing (the heterogenous nuclear ribonucleoprotein (hnRNP) family) and stimulating (the serine/arginine (SR) protein family) proteins for these splice modulators have been identified, both regulating alternative splicing indirectly (Tazi *et al*, 2005b; House and Kristen, 2008).

In conclusion, the removal of introns and/or exons has to be precise and complete, otherwise incorrectly or differently joined exons yield no mature and functional mRNA, or result in altered genetic messages with the potential of initiating malignant behaviour in previous normal cells.

## Alternative splicing and disease/cancer

Recent research has revealed that alternatively spliced products can be linked to various (genetic) diseases and also may play a role in cancer development (Wang and Burge, 2008). A systematic approach using large-scale sequencing and splicing-sensitive microarrays gave for the first time a view of the vast spectrum of alternative transcripts (Blencowe, 2006; Ben-Dov *et al*, 2008). One or more alternatively spliced exons can be found in transcripts of two-thirds of human genes. In general, the function of the encoded protein remains unchanged. However, some of the alternative protein products may display a malignant phenotype (Blencowe, 2006). Whether alternative splicing is the cause or result of malignant behaviour in cancer cells remains unclear. However, as alternative splicing affects most genes, it is likely that cell cycle control, signal transduction, angiogenesis, motility and invasion and the metastasis and apoptosis processes that are often impaired in cancer will be affected (Skotheim and Nees, 2007).

Extensive research indicates that several familial cancer syndromes are caused by somatic (point) mutations or single-nucleotide-polymorphisms (SNPs) occurring in splice sites. These changed splice-site signals may give rise to alternative splice variants of tumour suppressor genes such as *BRCA1*, *APC*, *p53, FHIT* and *LKB1* and the prostate cancer susceptibility gene, *KLF6* (see for a recent review Skotheim and Nees, 2007). Moreover, it has been shown that splice variants of H-RAS, the enzyme phosphatase PPR2C*γ*, CD44v3 (a cell surface glycoprotein), SPN1*Δ*N (a deletion mutant of snRNP-specific nuclear import adapter snurportin 1), RTVP-1 (in malignant gliomas) and CD44v6 (in various epithelial cancers) all favour tumourous behaviour in cells (Allemand *et al*, 2007; Barbier *et al*, 2007; Rino *et al*, 2007; Vela *et al*, 2007). The increasing number of known non-familial cancers that express alternatively spliced proteins, each of which may contribute to the malignant phenotype, provides additional impetus to search for agents that interfere with alternative splicing and may display an anticancer potential (Barbier *et al*, 2007).

Currently, three different approaches to target (alternative) splicing in cancer are explored. First, one may be able to modulate alternative splicing by identifying specific splice sites and directly or indirectly block their use. Alternatively, one may identify and characterise splice variant products (ie, proteins) that contribute to the malignant phenotype and determine how they differ from their normal protein counterparts. Structural differences may then be used to develop antibodies or small molecules that specifically target these proteins. A second approach may be the manipulation of splice modulators, for example, hnRNPs or SR proteins, hereby indirectly influencing splicing activity. A third approach is based on the simple notion that malignant cells in general have higher metabolic rates than normal cells, and consequently have increased splicing rates and are more prone to splice inhibitors or modulators. Targeting the spliceosome directly might therefore cause tumour inhibition at drug levels that do not affect normal cells.

The three strategies such as (i) splice-site modulation and targeting of variant proteins; (ii) targeting of splice modulators and (iii) spliceosome inhibitors are reviewed in detail below.

## Splice-site modulation and targeting of variant proteins

The delineation of splice sites used in alternative splicing or the functional characterisation of aberrant proteins expressed as a result of alternative splicing may provide the necessary insight for therapeutic interference. Specific splice-site mutations or proteins might (i) prove to be of predictive value and (ii) become potential prognostic markers (Yu *et al*, 2007), (iii) prove to be targets of future (anticancer) antibody-guided drugs (Orban and Olah, 2003; Heider *et al*, 2004; Xiang *et al*, 2007) or (iv) become a target for peptide receptor radionucleotide therapy. An example of the latter is a splice variant of the cholecystokinin receptor found in colorectal and pancreatic cancer that can be targeted by radionuclide therapy (Laverman *et al*, 2008). A disease in which an understanding of the alternative splicing process is used for therapeutic purposes is Duchenne's muscular dystrophy (DMD). Duchenne's muscular dystrophy is a neuromuscular disease caused by deletions/duplications or point mutations in the 2.4 Mb *DMD* gene, encoding dystrophin, causing disruption of the open reading frame (ORF). The induction of exon skipping, circumventing the mutated exon through intramuscular injection of carefully designed antisense oligonucleotides, corrects the ORF of dystrophin in *in vitro* cell lines, animal models and humans (van Deutekom *et al*, 2007). Another example concerns spinal muscular atrophy (SMA) caused by the deletion of *SMA1* gene and the inability of the remaining *SMA2* gene, which is virtually identical to *SMA1*, to compensate for the SMA1 loss, as its transcript lacks exon 7. Here, antisense oligonucleotides targeting an intronic splicing silencer (ISS-N1) may fully restore *SMN2* exon 7 inclusion (Singh, 2007).

If we indeed are able to create therapeutic approaches that correct the deleterious effects of splice variants by remodelling the (alternative) splice reactions in non-malignant diseases, it is conceivable that this approach might also be applicable in malignancies; Here, splice-site modulation would be a valuable concept for intervening in the expression of various oncogene and tumour suppressor gene mutations.

## Targeting of splice modulators

The SR proteins comprise a family of splice factors that modulate splice-site selection by mediating the interaction between pre-mRNA transcripts and the spliceosome. Phosphorylation of the serines in their arginine/serine (RS) domains regulates the intracellular localisations of SR proteins and their interaction with related domains of other splice factors or RNA. This consequently affects alternative splicing of pre-mRNAs. There are several kinases, for example, Clk/Sty, SRPK1, SRPK2 and topoisomerase I that are known to phosphorylate SR proteins. Several compounds have been reported that target these kinases, for example, diospyrin (Tazi *et al*, 2005a), indole derivatives (Soret *et al*, 2005) and indolocarbazole (NB506; Pilch *et al*, 2001), inhibit the kinase activity of topoisomerase I. Similarly, a benzothiazole compound displayed clear inhibitory effects on the activity of Clk1/Sty (Muraki *et al*, 2004). It was shown that exposure to these compounds modulates the splicing profile of several genes, and alters gene expression patterns, due to inhibition of SR protein phosphorylation. In future, drugs like these or derivatives thereof may be used for the therapeutic manipulation of alternative splicing.

## Spliceosome inhibitors

Recently, two chemically different microbial natural products with profound cancer cell inhibiting potential were found to target the spliceosome. Both compounds, the pladienolide derivatives (Kotake *et al*, 2007) and spliceostatin A (Kaida *et al*, 2007), appear to bind to SF3b, a subcomplex of U2 snRNP, which is an essential component of the spliceosome (Figure 2). SF3b together with SF3a enable U2 snRNP to associate with the branch point region near the 3′ end of the intron. The SF3b subcomplex consists of seven proteins such as the spliceosome-associated protein (SAP) 10, SAP14a, SAP14b, SAP49, SAP130, SAP145 and SAP155. The binding of pladienolide and spliceostatin A to SF3b causes an inhibition of the spliceosomal functions resulting in impaired splicing, and thus altered gene expression patterns.

Pladienolide compounds, in particular pladienolide B and D, displayed anticancer activity (Mizui *et al*, 2004; Kotake *et al*, 2007). E-7107 is a synthetic urethane derivative of pladienolide D with activity against tumour cell lines and human tumour xenografts (Iwata *et al*, 2004). Treatment of a drug screen panel of 39 cancer cell lines with pladienolides resulted in profound growth inhibition with greatest activity against breast and lung cancer cell lines. Comparison of the pladienolide sensitivity profile to that of other anticancer agents revealed that pladienolide has a unique and distinct antitumour spectrum. Additionally, a panel of six cancer cell lines made resistant against classic cytotoxic compounds such as camptothecin, etoposide, vincristine, 5-fluorouracil, doxorubicin and cisplatin were just as sensitive to pladienolide as their drug-sensitive parental cell lines. Although in most cell lines, the exact mechanism of drug resistance was not known, at least one cell line overexpresses P-glycoprotein (MDR1 and ABCB1) implying that pladienolides are not a substrate for this ubiquitous drug transporter (Mizui *et al*, 2004). Administration of variable doses of 2.5–10 mg kg^−1^ day^−1^ of pladienolide B for 5 consecutive days in six human tumour xenograft models, representing breast, ovarian, colon and prostate cancer, caused dose-dependent growth inhibition. Moreover, in the breast cancer BSY-1 xenograft, a clear tumour regression with no sign of recurrence for 36 days was noted (Iwata *et al*, 2004; Mizui *et al*, 2004; Kotake *et al*, 2007). The most prominent adverse effects were a reversible weight loss and dose-limiting haematopoietic toxicity.

By using differently tagged pladienolide derivatives as chemical probes, it was discovered that pladienolide interacts with SAP130 of the SF3b subcomplex. However, a partial interaction with additional components of SF3b (SAP145 and SAP155) cannot be ruled out (Kotake *et al*, 2007). Although the precise functions of SAP130, SAP145 and SAP155 within the U2 snRNP are not clearly defined, it is likely that the activity of SF3b and hence the U2 snRNP is modulated or impaired by pladienolide binding as indicated by the observed time and dose-dependent disturbance of *in vivo* splicing and the associated growth inhibition. Pladienolide treatment causes an accumulation of unspliced or incompletely spliced pre-mRNAs and gives rise to fewer and larger nuclear speckles and intranuclear sites where splice factors are stored. Similar phenotypes were observed when other spliceosome components like the U1 and U6 snRNAs were knocked down by antisense oligonucleotides (Kotake *et al*, 2007), or when the SF3b components such as SAP130, SAP145 and SAP155 were downregulated by siRNAs (Kaida *et al*, 2007).

FR901464 and its methylated derivative spliceostatin A also interact with SF3b, most likely by binding SAP130 or SAP155 (Kaida *et al*, 2007). Similar to the effects induced by pladienolide, splicing is partially inhibited causing pre-mRNA to accumulate. Whereas upon spliceostatin A treatment, most pre-mRNA species are retained in the nucleus, a subset of unspliced or partially spliced pre-mRNA is prematurely transported to the cytoplasm where it is translated into aberrant proteins. These observations strongly suggest that SF3b is not only involved in splicing but also in the retention of pre-mRNA. FR901464 and most likely spliceostatin A are potent anticancer agents affecting the proliferation of cancer cell lines (Kaida *et al*, 2007 and references therein), and inhibiting tumour growth in various xenograft models (Nakajima *et al*, 1996). The observation that cells exposed to FR901464, spliceostatin A and pladienolide showed a cell cycle arrest in G1 and G2/M phases and prompted a study of the involvement of cell cycle regulators, including cyclins, cyclin-dependent kinases (CDKs) and some positive and negative regulators of these proteins. It was found that cyclin A and CDK levels were unchanged, whereas p16 and p27, both negative regulators of CDKs levels, were increased. In addition, a C-terminally truncated p27 is expressed due to the translation of an aberrantly spliced p27 pre-mRNA. The truncated p27, however, is still capable of inhibiting CDK2 and may be partly responsible for the observed cell cycle arrest, and perhaps the antitumour activity (Kaida *et al*, 2007).

Clearly, binding of pladienolide and spliceostatin A to SF3b components interferes with the splicing process as well as surveillance mechanisms that are operational in the cell to prevent the synthesis of truncated proteins. On the basis of the promising preclinical data with respect to the potential anticancer activity of pladienolides B and D and E-7107 phase I dose-escalation trials with E-7107 are currently ongoing in Europe and the United States. Apart from determining the classic toxicity end points, extensive pharmacodynamic analyses are performed that may help to define appropriate biomarkers to assess the efficacy of these novel agents. The detailed analysis of drug-induced changes in gene expression patterns may give a deeper insight into the mode of action of these drugs and better define the beneficial effects for cancer patients.

## Conclusions

RNA splicing is an essential and complex cellular process, which is regulated and mediated by many different factors. Now that our understanding of the physiology of the spliceosome increases as well as our perception increases how (alternative) splicing may contribute to cancer, we hopefully can find ways to control and intervene with splicing, hereby preventing the expression of carcinogenic proteins. The pladienolide compounds and spliceostatin A that target the spliceosome may very well become the first representatives of a totally new class of anticancer agents.

## Figures and Tables

**Figure 1:**
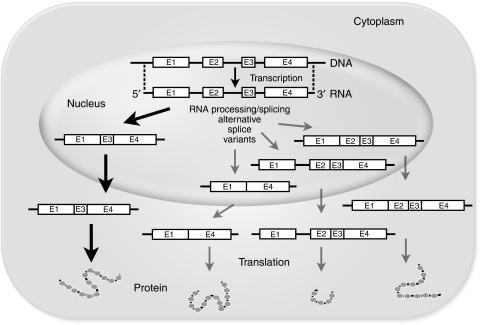
Schematic illustration of transcription, pre-mRNA processing/splicing and translation. A protein-coding gene is transcribed by RNA polymerase II into pre-mRNA. Subsequently, or already during transcription, non-coding introns are removed in a process called splicing, after which the mature mRNA is capped and polyadenylated and transported to the cytoplasm where it is translated into protein. Depicted is a gene consisting of four exons interspersed by three introns. Under normal splicing conditions, exons 1, 3 and 4 are linked together forming the vast majority of mature transcripts (bold black arrow). Alternative splicing may recombine gene elements into new configurations that may or may not display the activity or characteristics of the original protein. For example, alternative splicing reactions (grey arrows) can result in the acquirement of an extra exon (exon 2), in the skipping of exons (exon 2 and 3) or in an extra exon and part of the first intron through the use of a cryptic splice site in the first intron. In the latter case, a truncated protein may form due to the presence of a termination codon in the remaining intron sequence. Under normal circumstances, nonsense-mediated decay (NMD) is responsible for the degradation of mRNAs that have a premature stop codon to prevent the synthesis of detrimental truncated proteins. Note that the NMD is dysfunctional in cells treated with spliceostatin A leading to the expression of truncated proteins (see text for details).
